# Association of time under immunosuppression and different immunosuppressive medication on periodontal parameters and selected bacteria of patients after solid organ transplantation

**DOI:** 10.4317/medoral.22238

**Published:** 2018-04-24

**Authors:** Gerhard Schmalz, Lisa Berisha, Horst Wendorff, Florian Widmer, Anna Marcinkowski, Helmut Teschler, Urte Sommerwerck, Rainer Haak, Otto Kollmar, Dirk Ziebolz

**Affiliations:** 1 Department of Cariology, Endodontology and Periodontology, University of Leipzig; 2 Department of Pneumology, Ruhrlandklinik, West German Lung Center, University Hospital Essen, University Duisburg-Essen, Germany; 3 Department of General and Visceral Surgery, HELIOS Dr. Horst Schmidt-Kliniken, Wiesbaden, Germany

## Abstract

**Background:**

Aim of this study was to investigate the association of the time under immunosuppression and different immunosuppressive medication on periodontal parameters and selected periodontal pathogenic bacteria of immunosuppressed patients after solid organ transplantation (SOT).

**Material and Methods:**

169 Patients after SOT (lung, liver or kidney) were included and divided into subgroups according their time under (0-1, 1-3, 3-6, 6-10 and >10 years) and form of immunosuppression (Tacrolimus, Cyclosporine, Mycophenolate, Glucocorticoids, Sirolimus and monotherapy vs. combination). Periodontal probing depth (PPD) and clinical attachment loss (CAL) were assessed. Periodontal disease severity was classified as healthy/mild, moderate or severe periodontitis. Subgingival biofilm samples were investigated for eleven selected potentially periodontal pathogenic bacteria using polymerasechainreaction.

**Results:**

The mean PPD and CAL as well as prevalence of *Treponema denticola* and *Capnocytophaga species* was shown to be different but heterogeneous depending on time under immunosuppression (*p*<0.05). Furthermore, only the medication with Cyclosporine was found to show worse periodontal condition compared to patients without Cyclosporine (*p*<0.05). Prevalence of *Porphyromonas gingivalis, Tannerella forsythia* and *Fusobacterium nucleatum* was reduced and prevalence of *Parvimonas micra* and *Capnocytophaga species* was increased in patients under immunosuppression with Glucocorticoids, Mycophenolate as well as combination therapy.

**Conclusions:**

Time under and form of immunosuppression might have an impact on the clinical periodontal and microbiological parameters of patients after SOT. Patients under Cyclosporine medication should receive increased attention. Differences in subgingival biofilm, but not in clinical parameters were found for Glucocorticoids, Mycophenolate and combination therapy, making the clinical relevance of this finding unclear.

** Key words:**Immunosuppression, organ transplantation, periodontitis, periodontal bacteria.

## Introduction

Periodontitis (P) is an inflammatory disease with a multifactorial character (1). Although P is primarily caused by an opportunistic infection with potentially periodontal pathogenic bacteria, host response, general diseases (e.g. diabetes mellitus, rheumatoid arthritis, medication) and environmental factors are involved in the aetiopathogenesis of periodontal inflammation (1).

Considering the role of host response in this context, factors influencing the immune reaction of the host might be of relevance. Therefore, an immunosuppressive therapy could have an impact on periodontitis severity and progression via alterations in periodontal tissues, gingival overgrowth or ulceration and migration of junctional epithelium (2-5).

One special group receiving life-long immunosuppressive therapy consists of patients after solid organ transplantation (SOT). Recent studies showed high prevalence of periodontal diseases in this patient group (liver, lung, kidney), but did not investigate the immunosuppression separately (6-8). Consequently, up until now the clinical influence on periodontal parameters caused by immunosuppressive medication appears to be unclear. Especially the duration of immunosuppressive therapy, but also the specific drug as monotherapy or combination could be of relevance in this respect.

In development and progression of P, potentially periodontal pathogenic bacteria play an important role. It has been shown, that an ecological shift in the subgingival microbiota is a key component (9,10) Although their epidemiological relevance is discussed controversially, periodontal pathogenic bacteria as classified by Socransky *et al.* 1998 (11) are an important element of current understanding of pathogenesis of P. The influence of immunosuppression on periodontal bacteria is still unclear. Few available data for immunosuppressed patients after organ transplantation showed microbiological changes indeed (12-14). However, on the other hand differences between immunosuppressed transplant recipients and not immunosuppressed patients were not found in recent investigations (6,15). Similarly to periodontal condition, the influence of duration of immunosuppressive therapy and the specific drug as monotherapy or combination is unclear.

Therefore, the aim of this multicenter observational cross-sectional study was to investigate the association of the duration of immunosuppression and different immunosuppressive medication with periodontal parameters and selected periodontal pathogenic bacteria of immunosuppressed patients after SOT. For this, patients after transplantation of three different organs (kidney, liver, and lung) from previous studies by this working group should be investigated as one large cohort (6-8,15). It was hypothesized that the duration and form of immunosuppressive therapy would be associated with periodontal disease parameters, but not with prevalence of selected periodontal pathogenic bacteria.

## Material and Methods

This clinical, observational cross-sectional study was reviewed and approved by the ethics committee of the University Medical Center Göttingen, Germany (No. 43/9/07) and by the ethics committee of the University Hospital Essen (13-5689-BO). Research was conducted in full accordance with the World Medical Association’s Declaration of Helsinki. The patients were informed verbally, as well as in writing, about the study and provided written informed consent to participate.

- Patients

For this study, a group consisting of patients after solid organ transplantation from previous studies (6-8,15), has been composed. For this, previously defined, specific in- and exclusion criteria were used to select fitting patients for this study. Patients after liver and kidney transplantation in the Department of General, Visceral and Pediatric Surgery of the University Medical Center Göttingen (recruited between February to July 2012), and patients after lung transplantation from the lung transplant unit of the Ruhrlandklinik, Essen, Germany (recruited between February and October 2014) were investigated during their routine follow up visit.

SOT (kidney, liver, lung), irrespective of time under immunosuppression and regular subsequent appointment in one of the two transplant centers was the inclusion criterion for this study. The following exclusion criteria were formulated: age <18 years, presence of any additional infectious diseases, especially HIV, seizure and nervous disorder, as well as pregnancy. Furthermore, the inability to undergo complete oral investigation and toothlessness leaded to exclusion from the study.

- Recording of general medical conditions

Each patient was asked to complete a questionnaire to record the general medical conditions. Thereby, immunosuppressive and further medication, diabetes status, smoking habits (smoker = currently smoking, former smoker = currently non-smoking but smoked within the past 5 years and non-smoker = never smoking or non-smoking for at least 5 years), causal underlying disease for transplantation and time under immunosuppression were evaluated. Regarding time under immunosuppression patients were divided into five groups (0-1 year, 1-3 years, 3-6 years, 6-10 years and >10 years) for analysis. For the analysis of influence of different immunosuppressive medication, only the immunosuppressive drugs, which were taken by at least 15 patients, were included for analysis.

- Periodontal examination

All of the patients were examined under standardized conditions by an experienced dentist at the dental clinic of the University Medical Center Göttingen (kidney and liver), or in in the lung transplant unit of the Ruhrlandklinik, Essen, Germany (lung), respectively. One hour prior to examination, all patients received a single shot antibiotic prophylaxis (amoxicillin 2 g or clindamycin 600 mg at the discretion of the treating physician).

Firstly, a visual inspection of the oral mucosa and the gingiva to detect overgrowth was executed. Furthermore, the papillary bleeding index (PBI) has been performed to assess gingival inflammation (16). The bleeding of the marginal gingiva, which was evaluated using a periodontal probe (PCP 15; Hu-Friedy, Chicago, IL, USA), was the basis for the graduation of PBI score between 0 (no bleeding/inflammation-free gingiva) and 4 (profuse bleeding/severe inflammation). For the assessment of the periodontal status, periodontal probing depth (PPD) and clinical attachment loss (CAL) were measured on six measurement points per tooth using a millimeter-scaled periodontal probe (PCP 15; Hu-Friedy, Chicago, IL, USA). According to the AAP/CDC, periodontitis was classified into three categories: 1) severe periodontitis; 2) moderate periodontitis; or 3) no/mild periodontitis (17).

- Microbiological analysis 

Microbiological analysis was performed using subgingival biofilm samples from at least two up to a maximum of four of the deepest periodontal pockets. After placement of cotton rolls to avoid saliva contamination, supragingival biofilm was removed from the surface of the teeth investigated. After this, sterile paper tips were placed in the periodontal pockets for 20 seconds and all tips from the same patient were pooled together for analysis. Paper tips, which were contaminated with blood, were discarded. The microbiological analysis of selected potentially periodontal pathogenic bacteria was conducted using polymerase chain reaction (PCR). The following eleven selected potentially periodontal pathogenic bacteria were investigated: *Aggregatibacter actinomycetemcomitans* (*Aa*, detection threshold >102), *Porphyromonas gingivalis (Pg), Tannerella forsythia (Tf), Treponema denticola (Td), Prevotella intermedia (Pi), Parvimonas micra (PM), Fusobacterium nucleatum (Fn), Campylobacter rectus (Cr), Eubacterium nodatum (En), Eikenalla corrodens (Ec), Capnocytophaga species (Cs)*; detection threshold >103). All samples were analyzed in the clinical laboratory of the Department of Preventive Dentistry, Periodontology and Cariology, University Medical Center Goettingen, Germany. The semiquantitative detection of the bacterial colonization of the patients’ oral samples was executed using a commercial test system (Micro-IDentplus®-Test, HainLifescience, Nehren, Germany) according to the manufacturers protocol. In brief, amplification was performed with a 35-µl mixture of primers and dNTPs (Hain Lifescience, Nehren, Germany), 10.5 µl Mastermix (Qiagen, Hilden, Germany) and 5 µl of the DNA sample or 5 µl of water as a negative control, respectively. The amplification cycles were executed in a thermo cycler (Biometra, Goettingen, Germany). The hybri-dization was performed following the Micro-IDent plus protocol in a TwinCubator (Hain Lifescience, Nehren, Germany). Only the prevalence of the selected bacteria was considered for analysis.

- Statistical analysis 

Statistical analysis was performed using SPSS, version 24.0 (SPSS Inc., US). Kolmogorow-Smirnow-test was used to test the metric variables for their normal distribution. Thereby, none of the tested variables showed normal distribution. Accordingly, only non-parametric tests for non-normal distributed samples were performed. A comparison of more than 2 independent, non-normal distributed samples was executed using Kruskal-Wallis test, while categorical data were analyzed using Chi² or Fisher test, respectively. The significance level was set at *p*<0.05.

## Results

- Patients

A total of 169 immunosuppressed patients after organ transplantation with a mean age of 55.44 ± 11.10 years and an average time of 7.00 ± 5.72 years under immunosuppression could be included. The immunosuppressive medication, which were included for analysis were Tacrolimus, Cyclosporine, Mycophenolate, Glucocorticoids and Sirolimus, whereby 77% of patients received a combination of at least two different medications ( Table 1).

**Table 1:**
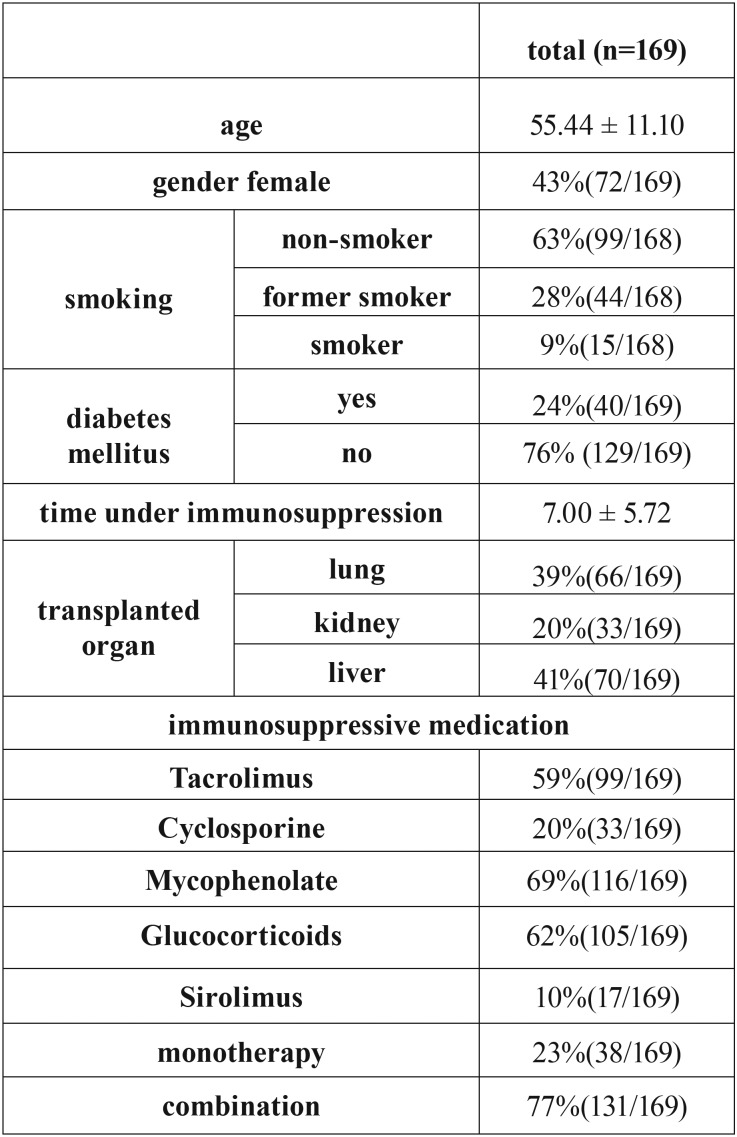
Characteristics of patients.

- Periodontal examination

Five percent of patients showed a gingival overgrowth, of which significantly more patients received medication with cyclosporine (63% vs. 37%; *p*<0.01). According to the classification of the AAP/CDC, 17% of patients showed no or mild periodontitis, while 56% were found to present a moderate, and 27% a severe periodontitis. Thereby, the number of remaining teeth was on average 17.77 ± 7.56. The periodontal findings are given in  Table 2.

**Table 2:**
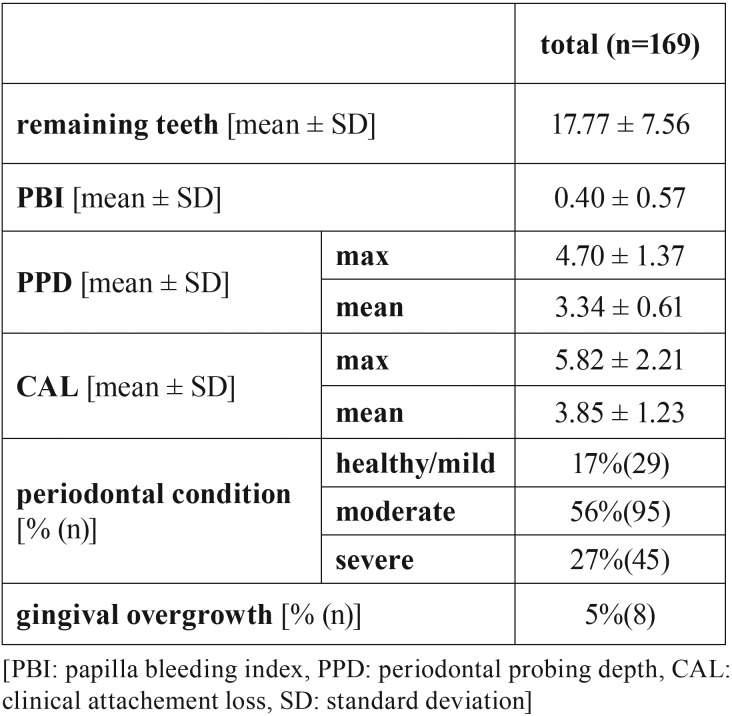
Periodontal findings of patients.

- Periodontal parameters depending on time under and form of immunosuppression

The mean PPD and mean CAL was shown to be different but heterogeneous depending on time under immunosuppression (*p*<0.05,  Table 3). In analysis of selected immunosuppressive drugs, cyclosporine was found to present higher mean PPD (*p*=0.04), mean and max CAL (*p*=0.05) as well as periodontal disease severity (*p*=0.02,  Table 4).

**Table 3:**
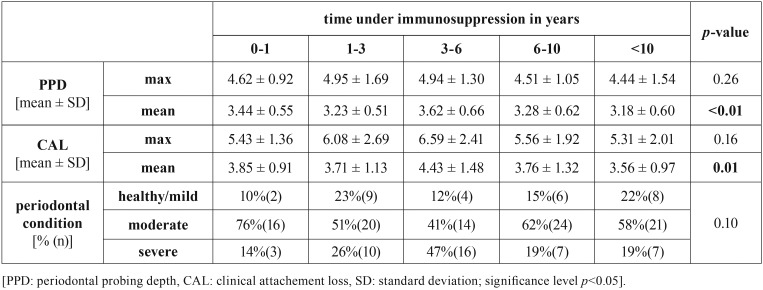
Periodontal findings depending on time under immunosuppression.

**Table 4:**
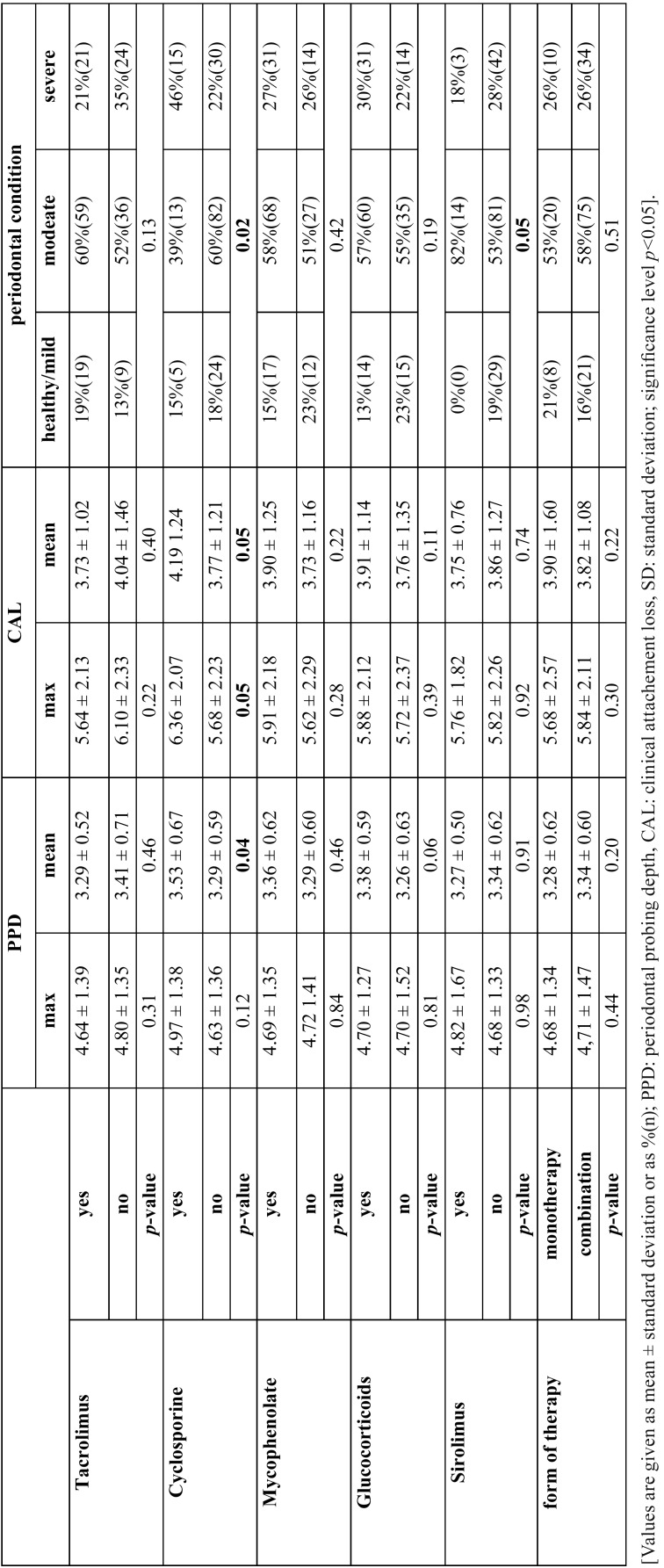
Periodontal condition depending on immunosuppressive medication.

- Periodontal bacteria depending on time under and form of immunosuppression

**Figure 1:**
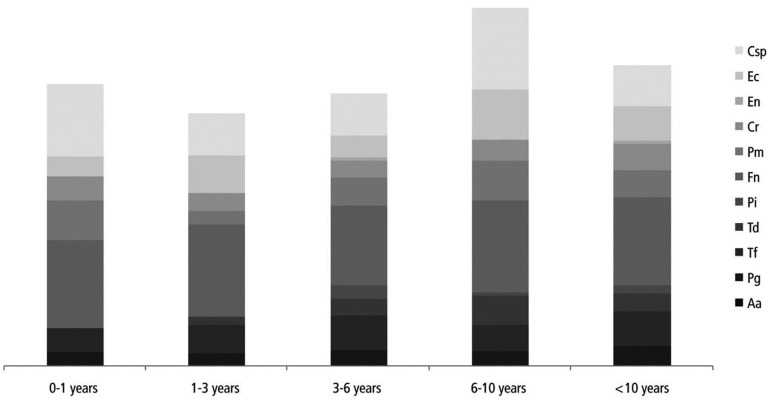
Prevalence of selected periodontal pathogenic bacteria depending on time under immunosuppression.

Out of the selected potentially periodontal pathogenic bacteria, for Td and Csp heterogeneous, but statistically significant differences in the prevalence depending on time under immunosuppression were found (*p*<0.05, figure 1).

**Figure 2:**
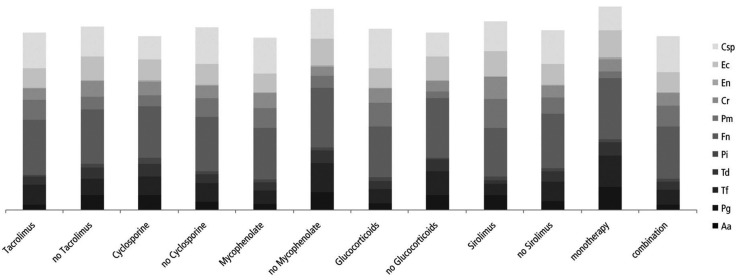
Distribution of selected periodontal pathogenic bacteria depending on immunosuppressive medication.

Patients under immunosuppression with Glucocorticoids showed significantly lower prevalence of Pg (*p*=0.03), Tf (*p*=0.04) and Fn (*p*=0.01) and higher pre-valence of Pm (*p*<0.01) and Csp (*p*<0.01) compared to patients without glucocorticoid medication. Also for combination vs. monotherapy, lower prevalence of Pg (*p*<0.01), Tf (*p*=0.01) and a trend for Fn (*p*=0.07) as well as a higher prevalence of Pm (*p*=0.03) and a trend for Csp (*p*=0.07) were found. Similar results were found for Mycophenolate with lower prevalence of Pg (*p*<0.01), Tf (*p*<0.01) and Fn (*p*=0.01) as well as a trend for higher prevalence of Pm (*p*=0.06). Furthermore, patients with Tacrolimus showed a lower prevalence of Pg compared to patients without Tacrolimus (*p*=0.01, figure 2).

## Discussion

- Main results: Time under immunosuppression was associated with both, the clinical periodontal findings (PPD and CAL) and prevalence of selected potentially periodontal pathogenic bacteria (*p*<0.05). However, these results were heterogeneous between the selected groups (0-1 year, 1-3 years, 3-6 years, 6-10 years and >10 years). Patients under medication with Cyclosporine were found to show worse periodontal conditions compared to patients without Cyclosporine, but further association of immunosuppressive medication with clinical parameters was not found. In contrast, medication with Mycophenolate, Glucocorticoids and combination of different immunosuppressive drugs was found to show lower prevalence of Pg, Tf and Fn, while the prevalence of Pm and Csp was reduced.

- Interpretation: To the best of authors’ knowledge, studies about the influence of different immunosuppressive medications are rare and partly outdated, what limits the comparability with the current study. The effect of immunosuppressive medication on periodontal condition has already been evaluated in existing review articles (2,18) The time under immunosuppression was shown to influence the periodontal parameters and subgingival plaque composition; however the evaluation was just between 90 days and 9 months (14,19,20) and in very small patient cohorts, including exclusively patients after kidney transplantation. No long-term results are available in the recent literature. As the current study is limited by the character of a cross-sectional study, the influence of immunosuppression in follow-up could not be assessed and therefore this can only be assumed based on the current study’s findings. An association with periodontal parameters and several bacteria was found indeed, but a clear tendency over the time e.g. a higher progression of periodontal destruction with increasing time under immunosuppression cannot be derived. Further study´s with a longitudinal design would be necessary to clarify the influence of time since immunosuppression on clinical and microbiological parameters.

The more promising approach is the difference of investigated parameters between different immunosuppressive drugs. Some, but partly outdated data are available regarding this issue. It has been shown, that immunosuppressive medication might reduce periodontal inflammation in patients after kidney transplantation (21). These results were discussed controversially, but a reduced periodontal inflammation in immunosuppressed compared to non-immunosuppressed patients appears probable (2,18). The current study did not investigate periodontal inflammation in particular, but the missing differences in all periodontal parameters of the medication groups except for Cyclosporine makes the clinical relevance and validity of the available studies unclear.

An aspect of potential clinical relevance could be the worse clinical periodontal parameters between patients with and without Cyclosporine. Cyclosporine is well known to be associated with gingival overgrowth (2,21), what is in line with the current study’s findings, even though only eight patients showed gingival overgrowth. However, the influence on periodontal destruction is discussed controversially based on animal studies (22,23). The gingival overgrowth might explain higher PPD in patients with Cyclosporine. However, in the current study only very few patients were found to present gingival overgrowth. Therefore, based on the clinical findings of the current study, an influence of Cyclosporine on periodontal destruction appears probable, what is contradictory to the recent investigations (22,23).

As a further finding, differences in the prevalence of several potentially periodontal pathogenic bacteria (Pg, Tf, Fn, Pm, Csp) were found in patients receiving Glucocorticoids, Mycophenolate as well as the combination of at least two immunosuppressive drugs. Thereby, a kind of shift can be observed, in which classical periodontitis associated pathogens as Pg, Tf and Fn are reduced in their prevalence, while the prevalence of other bacteria as Pm and Csp is increased. Only little literature is available, which exclusively investigated immunosuppressed patients after kidney transplantation (12-14). Thereby, the results of Vieira *et al.* show a lower prevalence of putative anaerobic bacteria in immunosuppressed kidney transplant recipients (13). This is in line with the current study’s findings. The subgingival biofilm is complex and associated with several environmental factors as well as the host response (24,25). As the “classical” periodontal pathogenic bacteria including Pg and Tf have the potential to penetrate gingival epithelium and influence the host response, it is discussed whether these bacteria need inflammatory mediators in the gingival crevicular fluid for nutrition (26,27). If it is considered that inflammation might be reduced under immunosuppressive medication, the environmental conditions in periodontal pocket could be less favorable for these bacteria in case of immunosuppression. Furthermore, Fn is important in the formation of periodontal pathogenic biofilm as it can bind a lot of other bacteria (28), and reduce oxygen levels, what improves the ecological conditions for anaerobe bacteria like Pg (29). Consequently, reduction of Fn due to immunosuppression resulting in reduction of Pg could be a further approach.

The combination of different immunosuppressive medication, but also the usage of Glucocorticoids and Mycophenolate could mean a strong immunosuppression, resulting in higher changes in subgingival biofilm. Nevertheless, this appears to have no influence on the periodontal burden of the patients in the current study, as it is similar between different medications. Therefore, clinical relevance of this finding is questionable.

- Strengths and limitations: This is the first study investigating the association of the duration of immunosuppression and different immunosuppressive medication with periodontal parameters and selected periodontal pathogenic bacteria of immunosuppressed patients after SOT. The large cohort and the comprehensive investigation is the major strength of the study. However, it is limited by the design as a cross-sectional study, which is not possible to provide strong findings on the longitudinal development. Furthermore, the patient group is heterogeneous as it includes different organs with different causal underlying diseases. However, patients after SOT are a difficult patient group and a high case number is hard to achieve within one singular organ. The missing assessment of periodontal inflammation (BOP) is a major limitation of the study, as it is only possible to illustrate the periodontal disease burden of the patients based on PPD and CAL. Furthermore, the investigation of only 11 selected potentially periodontal pathogenic bacteria is not able to illustrate the complex biofilm and must always take the clinical results into account (30). Accordingly, the separation of the subgroups according to their periodontal disease severity would provide stronger findings, but was omitted because of the low case number that the subgroups would have.

Besides these limitations, the study provides clinical findings of a large cohort of immunosuppressed patients and provides new findings regarding clinical and microbiological parameters of this patients group.

## Conclusions

Within the limitations of the study it has been shown that time under and form of immunosuppression is associated with clinical periodontal and microbiological parameters of patients under SOT. Thereby, Cyclosporine medication could be associated with worse periodontal condition, making a more intensive dental care of these patients necessary. Furthermore, a shift in subgingival biofilm in case of medication with Glucocorticoids and Mycophenolate as well as combination of immunosuppressive medication can be assumed. The clinical consequence and thus relevance of this finding appears unclear.
